# Prenatal Diagnosis of Intracardiac Tumors

**DOI:** 10.5935/abc.20160183

**Published:** 2016-12

**Authors:** Maria José Morais, Fátima Silva, Mónica Melo, Ana Carriço, Francisco Valente

**Affiliations:** Centro Hospitalar de Vila Nova de Gaia, Espinho - Portugal

**Keywords:** Heart Neoplasms/diagnosis, Ultrasonography/methods, Echocardiography/métodos, Rhabdomyoma, Tuberous Sclerosis, Prenatal Care

## Clinical Case

Fetal cardiac tumors are a rare finding in prenatal ultrasonography with an incidence of 1-2/10000.^1^ Rhabdomyomas are the most common tumors in intrauterine life, accounting for 60-86% of primary fetal cardiac tumors.^1,2^

According to some case series and mostly case reports, the prevalence of tuberous sclerosis associated with fetal cardiac rhabdomyoma is 50-80%, resulting in a perinatal mortality rate of 0-100%.^3^

The authors present the images of a clinical case of a 26-year-old pregnant woman, with depressive syndrome and without other relevant medical past or familial history, referred at 21 weeks of gestation to echocardiography examination due to the detection of intracardiac tumors in the morphologic ultrasound. The examination revealed the presence of two homogeneous, smooth-surfaced masses in the left ventricle. At 28 weeks one more tumor was detected in the right ventricle. There was no obstruction of left or right ventricular outflow. Heart rhythm was normal. There was no evidence of pericardial effusion or ascites. Other visceral tumors were not observed on focused scanning or magnetic resonance imaging. Genetic testing was performed and revealed a normal feminine karyotype without mutations in the TSC1/TSC2 genes. The authors present the images.

**Figure 1:**
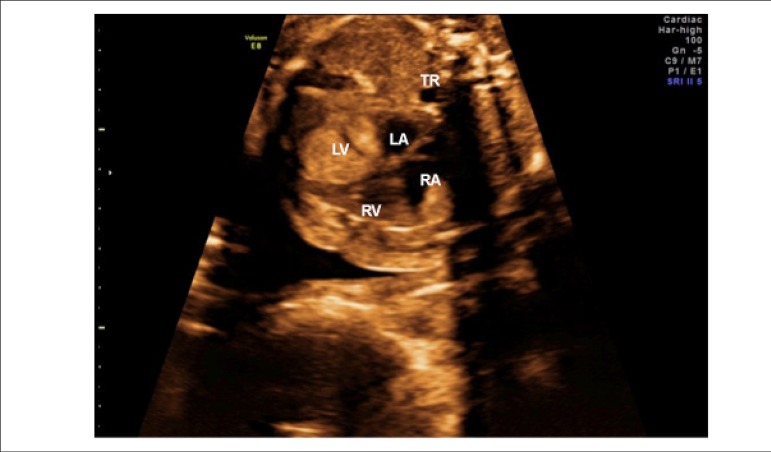
Ultrasound images at 21 weeks of pregnancy showing homogeneous, smooth-surfaced masses in the left ventricle. LA: left atrium; LV: left ventricle; RA: right atrium; RV: right ventricle; TR: trachea.

## References

[1] Geipel A, Krapp M, Germer U, Becker R, Gembruch U (2001). Perinatal diagnosis of cardiac tumors. Ultrasound Obstet Gynecol.

[2] Isaacs Jr H (2004). Fetal and neonatal cardiac tumors. Pediatr Cardiol.

[3] Chao AS, Chao A, Wang TH, Chang YC, Chang YL, Hsieh CC (2008). Outcome of antenatally diagnosed cardiac rhabdomyoma: case series
and a meta-analysis. Ultrasound Obstet Gynecol.

